# Low-temperature argon plasma jet with cascading electrode technique for biological applications

**DOI:** 10.1038/s41598-022-21664-9

**Published:** 2022-10-11

**Authors:** Pourya Seyfi, Maryam Keshavarzi, Saeed Zahedi, Ahmad Khademi, Hamid Ghomi

**Affiliations:** 1 Faculty of Mathematics, Physics and Informatics, Comenius University, Bratislava, Slovakia; 2 Laser and Plasma Research Institute, Shahid Beheshti University, Evin, Tehran, 1983963113 Iran; 3 Department of Mechanical and Biosystems Engineering, Faculty of Agriculture, Tarbiat Modares University, Tehran, Iran

**Keywords:** Applied physics, Plasma physics

## Abstract

In this study, the design, performance, and characteristics of a low-temperature argon plasma jet with cascading electrode technique (APJCE) are presented. APJCE is designed based on a tip-ring structure with a cascading ring. The effect of plasma jet driven by repetitive high-voltage microsecond pulses in APJCE structure was measured qualitatively in local surface temperature detection system. Then, by applying the generated plasma jet to biological surface and measuring and characterizing the electrical parameters, we obtained a plasma jet, which is electrically and thermally in the cold plasma regime. Simulation of the electric field distribution in the nozzle also yielded similar results to the experimental results. Finally, by cascading electrodes, we guided the plasma column to the nozzle output so that the plasma temperature within four centimeters of the nozzle output is 37 °C. The resulting plasma jets were studied by atomic emission spectroscopy and the intensity of the spectral lines of the atmospheric argon plasma jet spectra was obtained as a final experimental result at the output.

## Introduction

In the last decade, the use of new sciences and technologies and the application of these sciences in various industries such as agriculture, nanotechnology, nutrition and medicine have been considered by researchers, physicians and industrialists in the world^1–5^. One of the most important of these sciences is plasma and its essential applications, which have been very important in the development of science^3–7^. The use of plasma properties in surface activation, hydrophilicity and hydrophobicity, ozone generation, air pollution reduction, treatment of diabetic wounds, skin repair, treatment of cancerous tumors and numerous other applications is possible by using various plasma generation structures^4–13^. In the meantime, the use of plasma instruments is of particular importance due to their use in the synthesis of bio-surfaces as well as clinical tests^4^. Plasma jet is one of the most essential plasma instruments used in this field^12–16^. Argon and helium plasma jets with different gas combinations are the most important instruments used in the area of bioplasma^13–17^. To plasma jets generate, various jet nozzle structures and power supplies with different electrical parameters can be used^17,18^. Structures such as tip, ring, tip-ring, ring-ring are used depending on the experimental conditions and parameters that are desired^17,18^. Also, variations in power supply parameters play a vital role in the results of plasma treatment^17–19^. The use of high voltage nanosecond pulses, high voltage pulses in radio and microwave frequencies, mixed electric field^18^, kHz plasma jets as a very developed class of plasma devices and variations in pulse width and applied voltage have been among the most critical parameters that have been considered by researchers in this decade^14,19^. Another of the most fundamental variable parameters of plasma jets is gas and gaseous compounds used to generate plasma^14–19^. The generation of helium plasma jets is very easy due to the inherent properties of helium gas and plasma jets can also be generated by exciting a weak electric field^16–19^. But there are challenges to generating argon plasma jet that can be used in clinical experiments^4–15^. Creating streamers in the plasma column causes micro-discharge on the target and micro-discharge causes electric shocks on the target^20,21^. As a result of the micro-discharge colliding with the target surface, the surface temperature locally deviates significantly from the cold plasma temperature and damages the surface. Methods have been proposed by researchers to solve this problem. Using the microwave frequency as a source of plasma generation is one of the methods that generates low-temperature argon plasma jets^14^. But the design and construction of nozzles, waveguides and microwave frequency sources have complexities that make access to this plasma source difficult for all researchers^14^. In this paper, we present a structure that can be used to ignite argon plasma jets with biological applications by medium frequency high voltage pulses.

## Result

**Figure 1:**
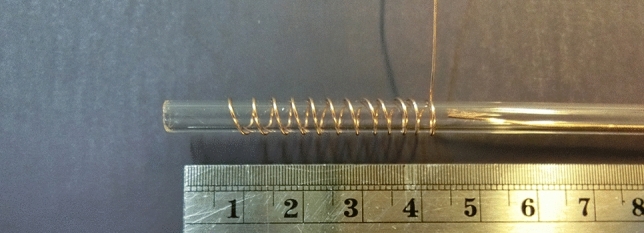
Jet nozzle with tip-ring method (cascade ring).

**Figure 2:**
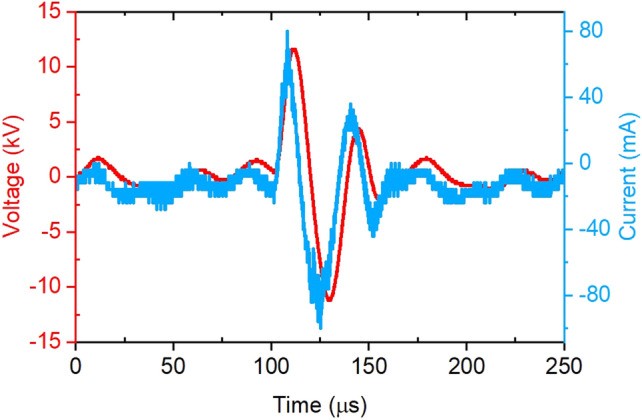
The voltage and jet current waveforms.

**Figure 3:**
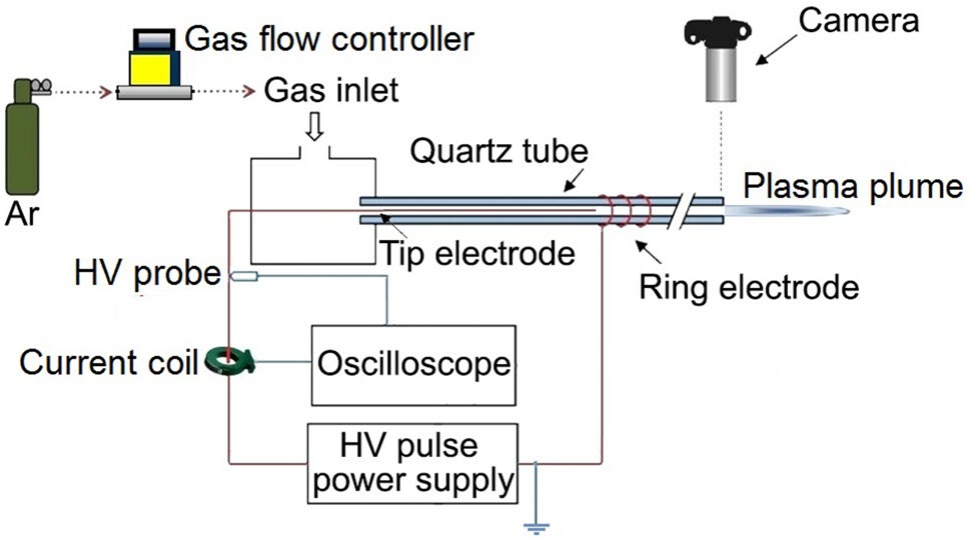
The experimental schematic of APJCE set-up and measuring instruments.

Figure 1 shows the actual image of the structure of the jet nozzle with the tip-ring method and with the cascade ring. This nozzle consists of a quartz tube with a diameter of 5 mm and a thickness of 0.8 mm. The tip-ring electrodes are made of copper wire with a diameter of 0.3 mm and are configured in different structures. A high voltage pulse power supply has been used to ignite argon gas in the jet nozzle and generate plasma jets. Figure 2 shows the pulse voltage and current diagram of the power supply used. High voltage pulse with an amplitude of 12 kV (Peak) and a pulse repetition frequency of 6 kHz is applied to the nozzle. Figure 3 shows the experimental schematic of APJCE set-up and measuring instruments. The following equations are used to calculate the average electrical power applied to the plasma jet. Where *P*_*ave*_ and *P*(*t*) are the average and time-dependent electrical power, respectively, *E*_*pulse*_ is the energy per pulse, *V*(*t*) and *I*(*t*) are the time-dependent voltage and current, respectively. Average electrical power is assumed to be constant (20 W) in all measurements and treatments.

1 $$ P \left( t \right) = V \left( t \right) \times I \left( t \right) $$

2 $$ E_{pulse } = \mathop \smallint \limits_{0}^{T} P\left( t \right)dt $$

3 $$ P_{ave} = \frac{{E_{pulse} }}{T} $$

**Figure 4:**
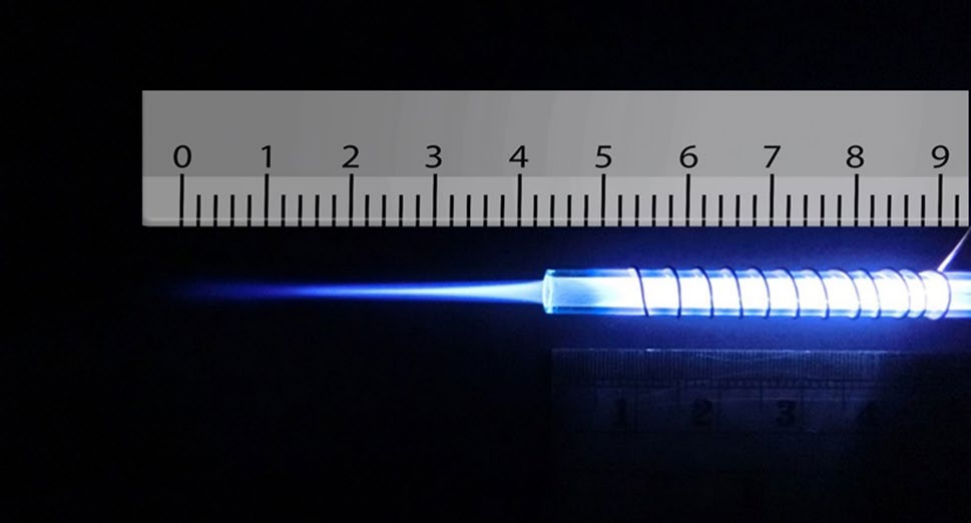
Argon plasma jet with APJCE structure.

**Figure 5:**
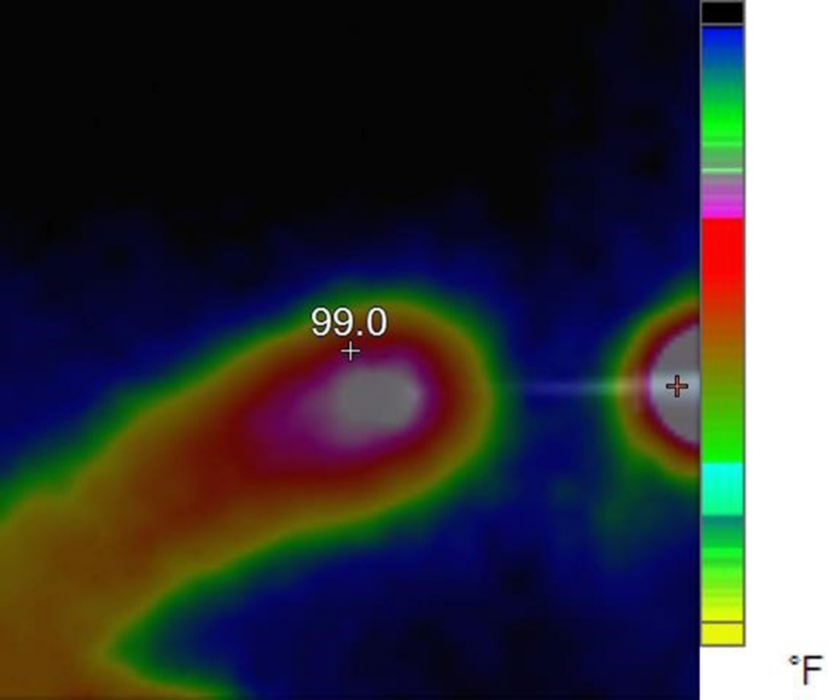
Thermal image of APJCE colliding with finger.

**Figure 6:**
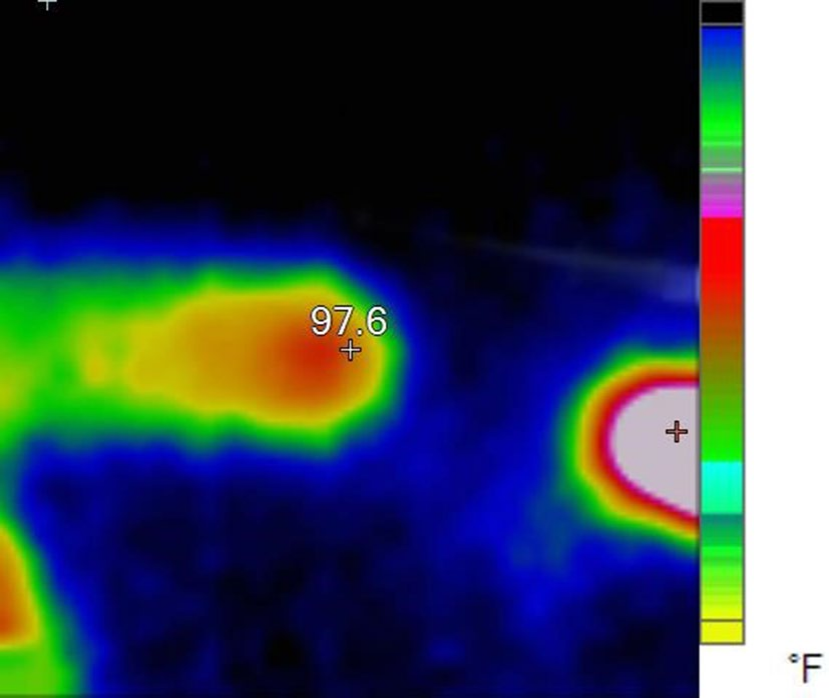
Thermal image of the finger as a reference.

**Figure 7:**
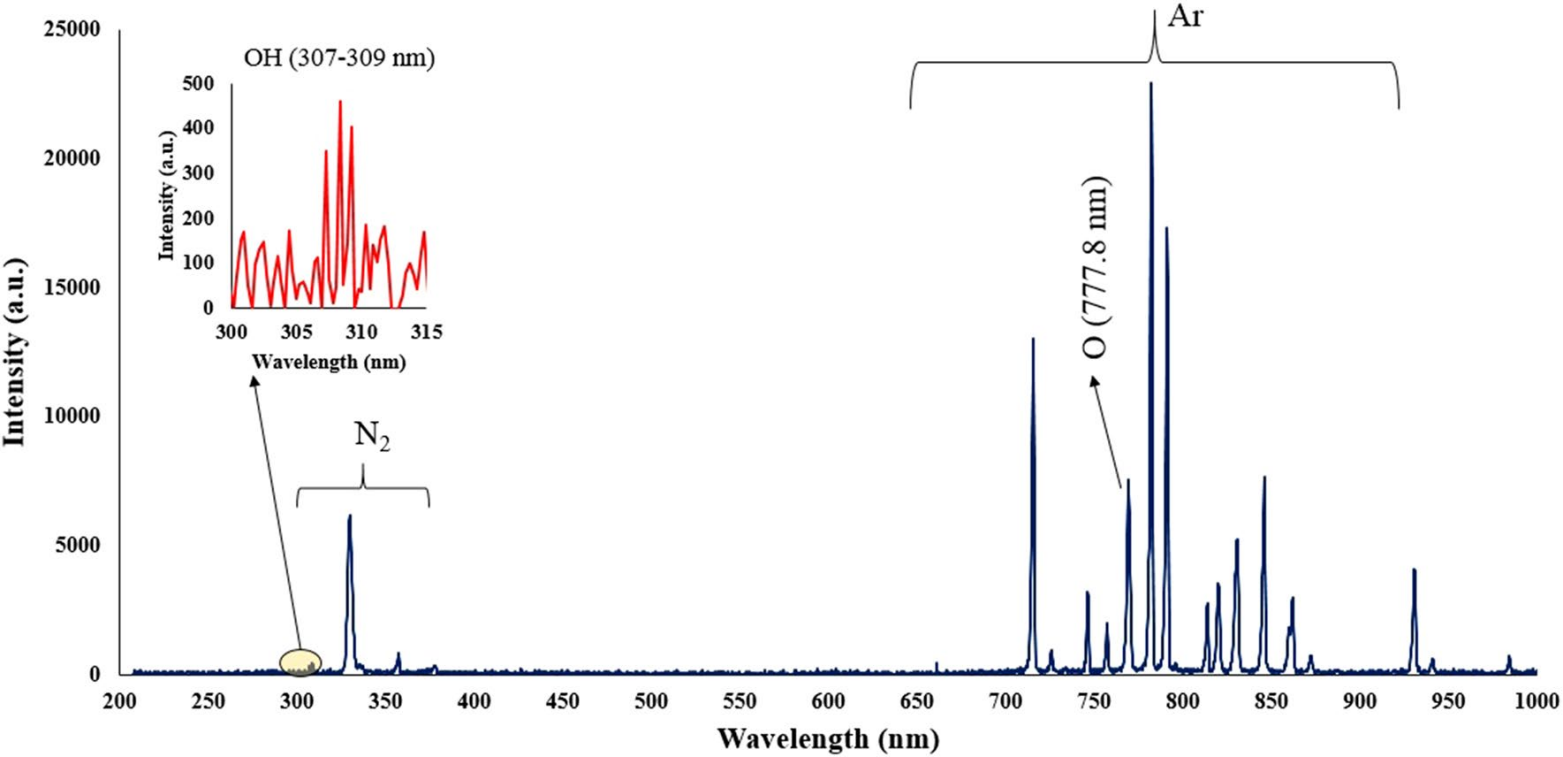
The OES of plasma jet (APJCE) at 1 cm away from the jet tube.

**Figure 8:**
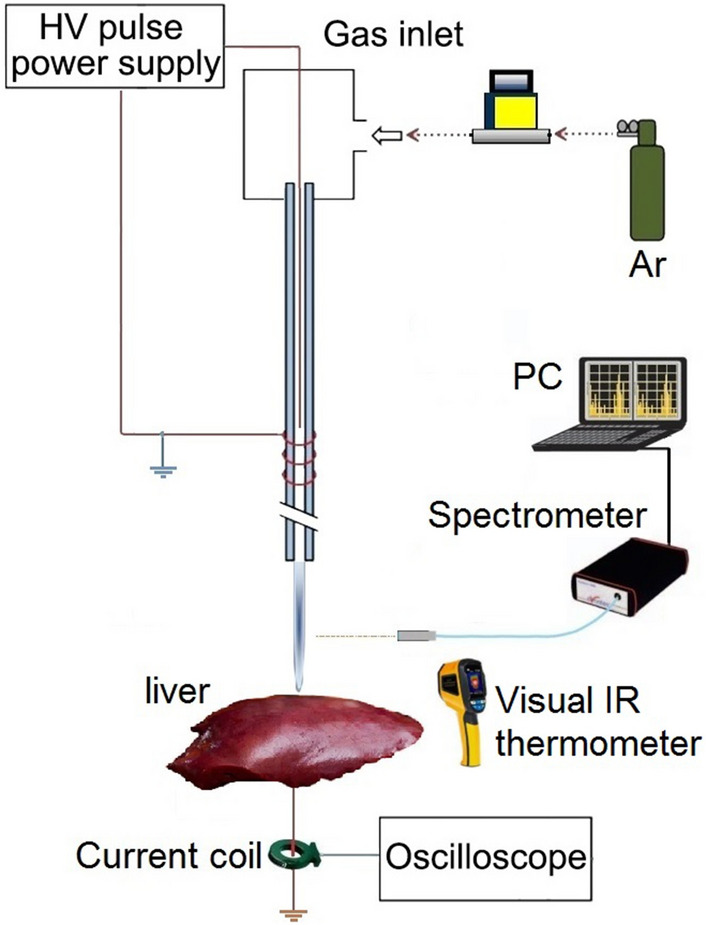
Experimental schematic of APJCE Irradiation with surface and measurements of electric shock and thermal effects results.

**Figure 9:**
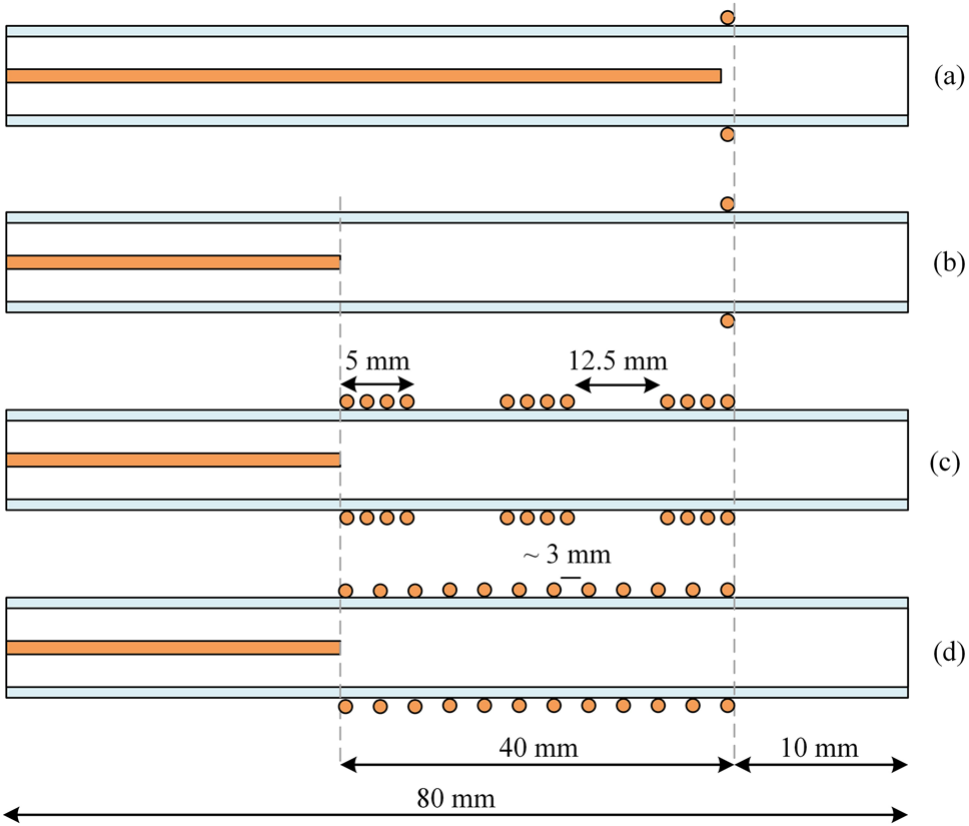
Different structures of plasma jet nozzles, (**a**) Tip-Ring1, (**b**) Tip-Ring2, (**c**) Tip-Ring-Cascading1, (**d**) Tip-Ring-cascading2.

Now we want to examine the experimental results. In the experimental conditions, we obtained the plasma jet of Fig. 4 with 12 turns of the cascade ring electrode. As shown in Fig. 4, the plasma plume is guided approximately 4 cm out of the jet nozzle outlet. In this structure, a 12-turn ring electrode is connected to the jet nozzle at the intervals reported in Fig. 9. The output obtained is the most optimal result compared to other structures, which we will analyze and compare in the following. But at this stage the APJCE is analyzed for local temperature and electric shock. The temperature distribution on the target surface was measured by Fluke VT04A Visual IR Thermometer. Thermal image Fig. 5 shows the thermal effects of a plasma jet colliding with a finger. The thermal image in Fig. 6 was used to compare the temperature in which the plasma jet did not collision the finger. This comparison shows that the plasma jet increases the local of collision temperature by only 1 °C. Thermal images show that the surface temperature after contact with the plasma has risen from 36 °C to 37 °C and this temperature is in the range of cold plasma. Atomic emission spectroscopy has been used to evaluate the accuracy of APJCE performance and plasma parameters. The intensity of the species in the plasma jet can be seen in Fig. 7. To investigate possible electric shocks, we used a biological target with approximate characteristics to the human body. For these experiments, the liver is used, as you can see in the structure in Fig. 8, to perform experimental tests of electric shocks caused by argon plasma jets and surface temperatures in various structures. The nozzle outlet distance from the target surface is 2 cm in all experiments. The thickness of the liver in these experiments is 25 mm. The impedance of the target plays a very important role in the collision behavior of the plasma jet on the surface of the target, but in this research, the goal is to design a plasma jet that works in optimal conditions without specifying target conditions^22^.

The nitrogen molecules, argon, OH and oxygen atoms spectral lines are observed in Fig. 7. The emission spectrum of the plasma jet obtained in this study is similar to the emission spectrum of the argon plasma jet at atmospheric pressure^23^. In this plasma jet emission spectrum, high-intensity peaks were related to excited argon species observed in the range of 700–955 nm wavelength. The atomic emission spectrum of oxygen O* was also observed at 777 nm. The lowest intensity peaks correspond to the OH spectrum in the range of 307 to 309 nm. Nitrogen exciting species were observed in the range of 310 to 430 nm. At this stage, other structures are investigated and finally compared. Figure 9 shows the different structures we have used for comparison. This figure is a cross-section of various structures of plasma jet nozzles.

**Figure 10:**
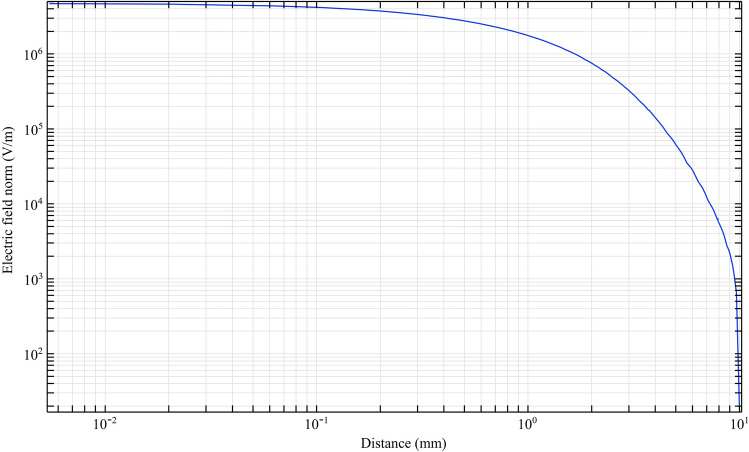
Simulation of electric field from tip electrode to end of plasma jet nozzle based on nozzle Fig. 9a.

**Figure 11:**
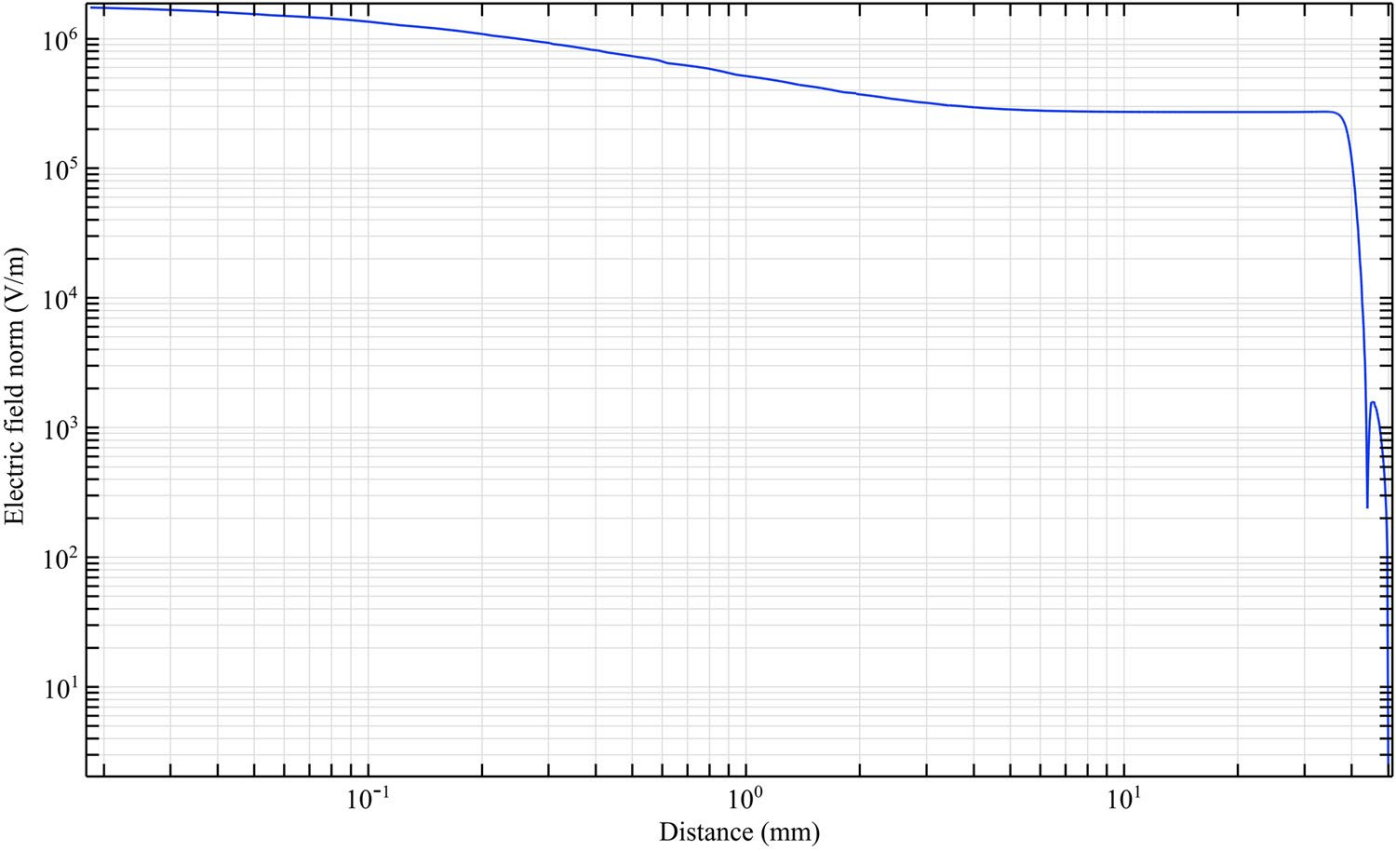
Simulation of electric field from tip electrode to end of plasma jet nozzle based on nozzle Fig. 9b.

**Figure 12:**
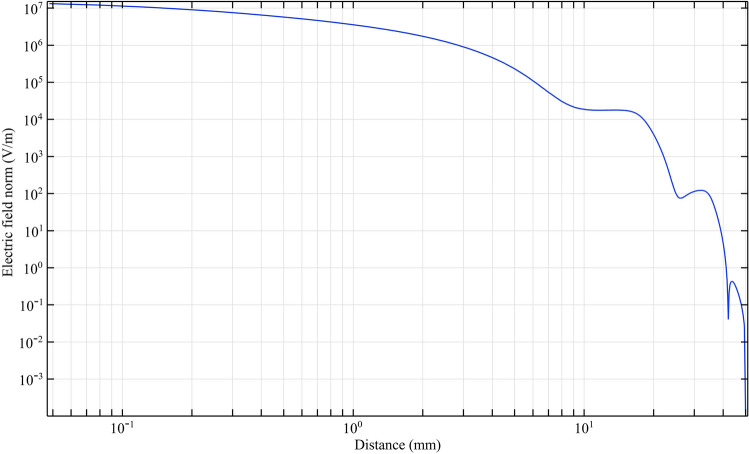
Simulation of electric field from tip electrode to end of plasma jet nozzle based on nozzle Fig. 9c.

**Figure 13:**
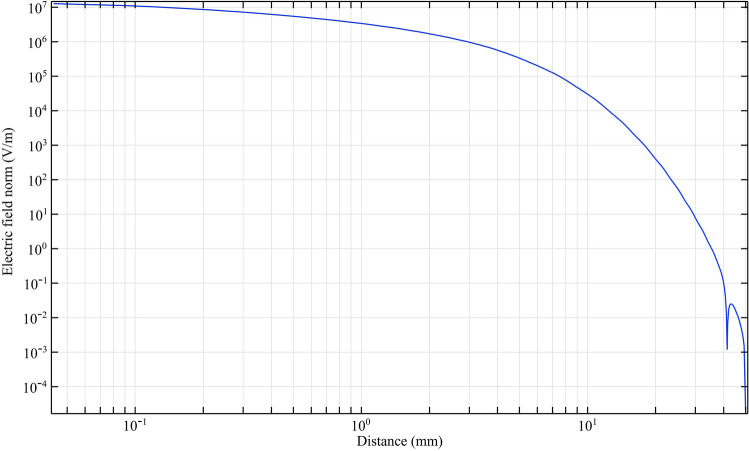
Simulation of electric field from tip electrode to end of plasma jet nozzle based on nozzle Fig. 9d.

**Figure 14:**
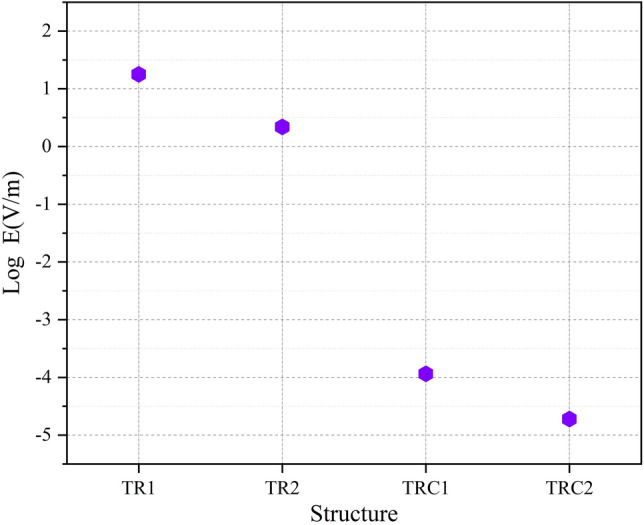
Comparison of electric field at the end of the tube in different structures of plasma jet nozzles.

With equal electrical conditions, we study the plasma jet and its performance in different structures. To deeply understand the performance of different nozzles, we first simulate the performance of the nozzles according to the nozzle structure in Fig. 9. According to this simulation, the average laplacian electric field from the tip to the end of the jet tube is calculated and plotted. The side view of the 2D simulation of average laplacian electric field of the plasma jet nozzle in the direction of the nozzle length is shown in Figs. 10, 11, 12, 13, 14. The laplacian electric field propagated in a quartz tube can be calculated based on the following equations:

4 $$ E = - \nabla \phi $$

5 $$ \nabla^{2} \phi = \rho $$

6 $$ D = \varepsilon_{0} \varepsilon_{r} E $$

7 $$ \nabla \cdot J = - \frac{\partial \rho }{{\partial t}} $$

8 $$ J = \sigma E + \frac{\partial D}{{\partial t}} + J_{e} $$

The parameters in the formulas refer to the following definitions.

*E *is the laplacian electric field intensity, $\phi$ is the electric potential, *ρ* is the electric charge density, *D *is the electric displacement, $\varepsilon_{0}$ is the permittivity of free space, $\varepsilon_{r}$ is the relative permittivity, *J *is the total current density, *σ* is the electrical conductivity and, *J*_*e*_ is the externally generated current density^24,25^. The boundary conditions for the electric potential are considered as follows and the calculations are performed accordingly. In the definition of time-dependent variables, the applied voltage to the tip electrode is defined as $\phi_{t} = V_{1} \left( t \right)$ and oscillates according to Fig. 2, and the applied potential to the ring electrode is defined as $\phi_{r} = 0$. Boundary conditions at the interface between the two media are expressed as follows:

9 $$ n \times \left( { E_{1} {-}E_{2} } \right) = 0 $$

10 $$ n \cdot \left( { D_{1} {-}D_{2} } \right) = \rho $$

11 $$ n \cdot \left( { J_{1} {-}J_{2} } \right) = - \frac{\partial \rho }{{\partial t}} $$

In the above equations n is the normal surface vector. The simulated laplacian electric field between the tip electrodes and end of plasma jet tube, based on the above-mentioned equations and boundary conditions, was performed and the results are shown in Figs. 10, 11, 12, 13. The average value of the electric field across the tip and outlet boundaries is given by

12 $$ \left( {norm E} \right)_{ave} = \frac{1}{T} \times \frac{1}{D}\smallint \smallint E\left( {s,t} \right) dsdt $$

which T is period time, D is arc length, and s is integral orientation.

By studying the diagram in Fig. 10, we can see the decreasing trend of the electric field from the tip to the end of the jet nozzle. The decrease rate of the electric field in Fig. 11, decreases with a different trend. This variation is due to the distance between the tip electrode and the ring electrode. In the Fig. 12 diagram, there is a fluctuation in the electric field. The fluctuation is caused by the structure of the ring electrode. Finally, in Fig. 13, the fluctuations in the previous structure are eliminated. But the important point is that while the trend of electrical field variations in all structures has a decreasing trend, the average electric field at the end of the jet tube is different in four structures. The variations mentioned above can be seen in Fig. 14. As can be seen in the diagram, in the two structures TR1 and TR2, the electric field at the end of the jet tube have a higher intensity than the structures TRC1 and TRC2.

**Figure 15:**
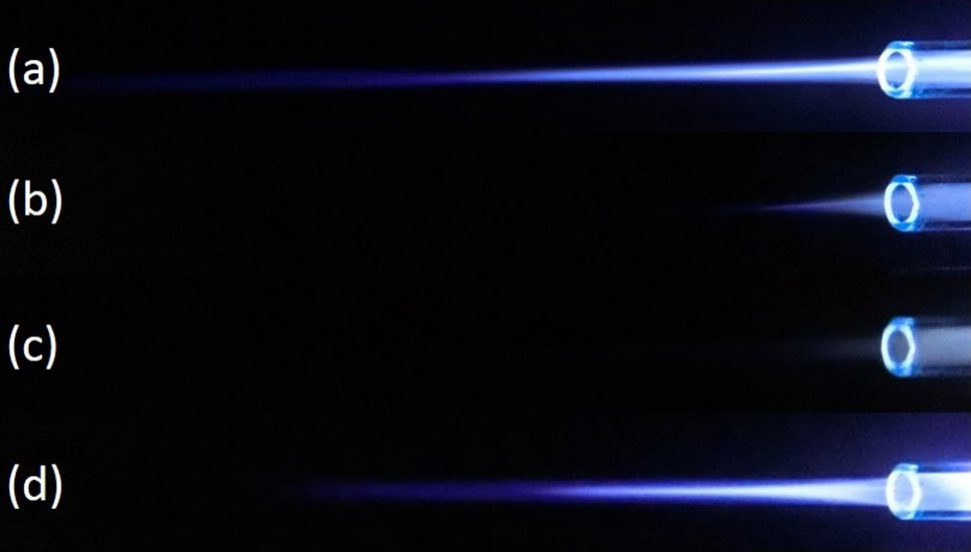
Comparison of plasma jet lengths according to the structures of Fig. 9.

**Figure 16:**
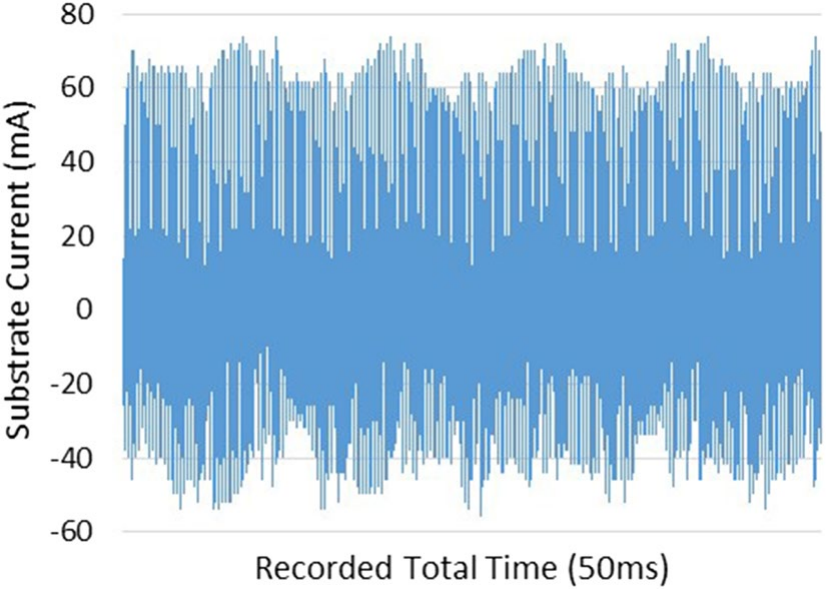
Substrate current, while the TR1 plasma jet collides the target.

Laplacian electric field is applied by the high voltage between the two electrodes along the length of the tube. Of course, one of the most main principles of plasma jet formation and propagation is the electric field of the space charge caused by the ionizing wave. But in this investigation, the Laplacian electric field as an important parameter that shows the average distribution of the electric field is separately investigated and compared^26–33^. In this step, we examine the simulated structures experimentally and then compare them with each other. The results are reported in Fig. 15. The results of plasma jet length show that we can have acceptable jet length in both Tip-Ring and Tip-Ring-Cascading structures. In TR1 structure, the maximum length of plasma jet with a length of more than 5 cm is obtained. After TR1 structure, TRC2 with a plasma jet length of more than 4 cm is obtained. The TRC2 structure has the longest plasma jet length after the TR1 structure. In the TR2 structure, a plasma jet is given out with a length of 1 cm from the tube outlet. But in the structure of TRC1 the plasma jet is very weak and the plasma column is not formed properly at the outlet of the nozzle tube. Comparison of simulation results and experimental results in TR1, TR2 and TRC1 can essentially confirm each other's final results. But in TRC2, unlike the lowest electric field at the end of the jet tube, a 4 cm length plasma jet is obtained at the outlet. Creating proper symmetry and distance in the cascade electrode rings causes the plasma column to be transferred from the tip electrode to the end of the jet tube and then propagated at a suitable length out of the jet nozzle. Asymmetry in the ring electrode impairs the performance of the cascade electrode. TRC1 represents an asymmetric cascade electrode that failed to properly transfer plasma to the outlet, and the plasma column in the nozzle is greatly damped, and at the nozzle output we see a weak column of the plasma afterglow. At this step, plasma jets in TR1 and TRC2 structures, which are acceptable structures in terms of plasma jet length, were subjected to experimental tests of electric shock and surface thermal degradation. Figure 16 shows the total electric current caused by the collision of a plasma jet with the surface. This electric current is related to the structure of TR1 and is measured according to the schematic in Fig. 8.

**Figure 17:**
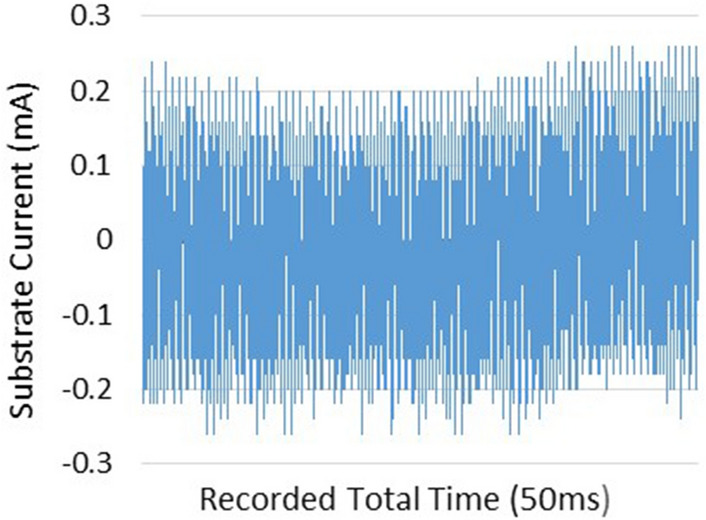
Substrate current, while the TRC2 plasma jet collides the target.

**Figure 18:**
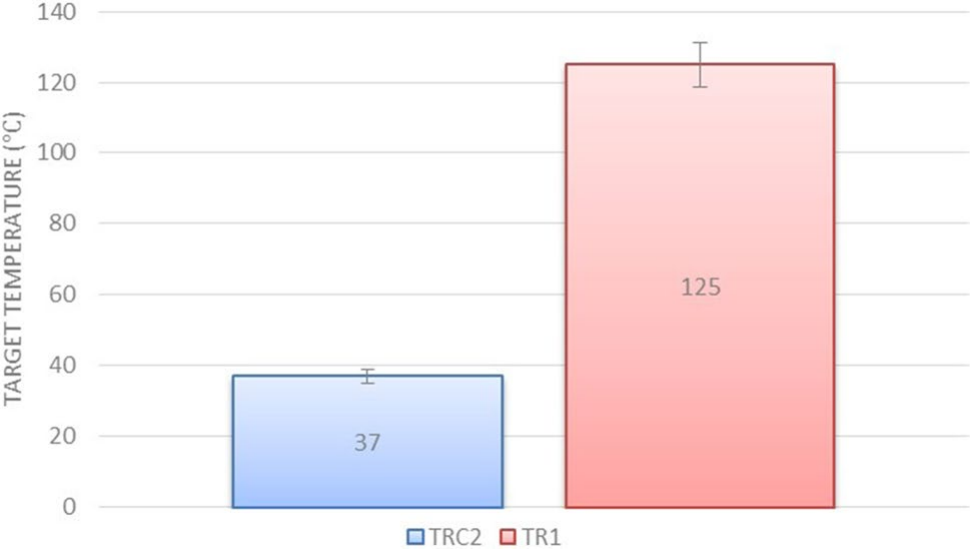
Plasma jet collision zone temperature with target surface in TR1 and TRC2 structures.

As shown in Fig. 16, the peak range of electric current pulses is in the range of 60 mA, and this range is in the scope of the pulse power supply current. These conditions indicate that the plasma column could not reach the acceptable ohmic resistance to prevent electric shocks. In Fig. 17, however, the peak range of electric current is in the range of 200 μA. By investigating the electric current of the substrate in the TRC2 structure, it can be concluded that in this structure, in terms of the electrical circuit, the plasma generation unit, ie the high voltage power supply, is isolated from the generated plasma jet. In this structure, electric shock is not applied to the target and only the plasma jet collides the surface with all the species generated. Finally, the temperature of the area where the plasma jet collides with the surface is measured and compared in two structures. The results of temperature measurements are reported in Fig. 18. The results clearly show that plasma jets generated with TR1 structure can cause severe thermal degradation of biological surfaces. But in comparison, if we use the TRC2 structure to generate plasma jets, the surface temperature will increase by only 1 °C compared to room temperature. Finally, we obtain a cold argon plasma jet capable of treating biological surfaces.

## Summary

Based on the presented results, a new method for generating argon plasma jets for biological applications has been proposed. With the new structure of the jet nozzle and the design of the new electrode structure, we were able to achieve a plasma jet without electric shock and thermal degradation of the surface in a simple yet very precise method. Generation and transfer of plasma jet by cascading electrode method to the structure allow, the output plasma jet to be electrically isolated from the source of plasma generation.

## Methods

**Figure 19:**
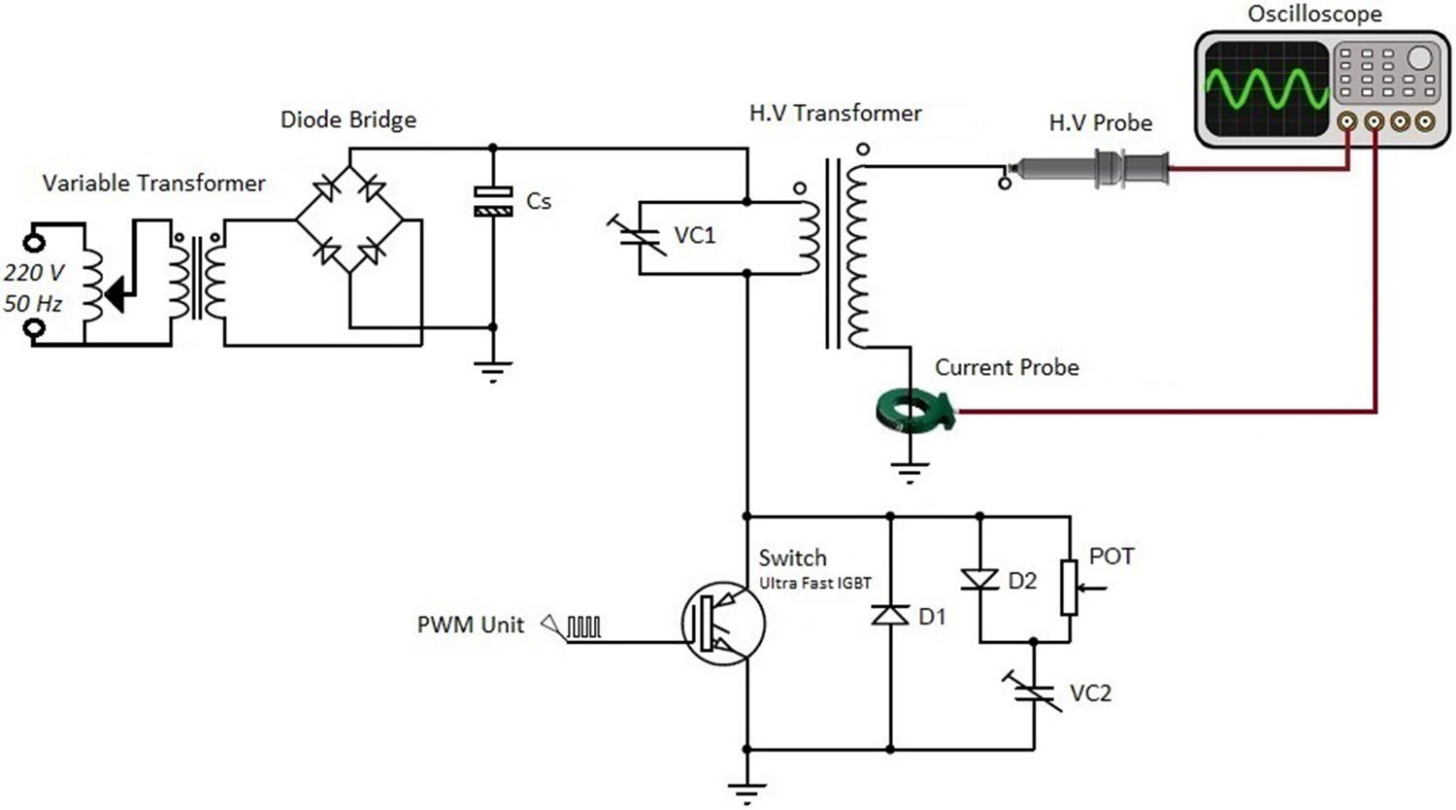
Pulsed high voltage power supply circuit.

A jet nozzle structure is designed with two electrodes, the electrode inside the tube as the igniter and the outer electrode as the controller of the plasma column. The inner electrode is designed as a tip and the outer electrode is designed as a cascading ring. This electrode is spirally wrapped around the tube in all three cases. The power supply used is a high voltage resonant pulse generating circuit designed in flyback mode. Figure 19 shows the circuits’ used^34^. The mass flow controller are employed to provide constant Ar flow (99.999%), which Located in the path of the argon bottle and the gas inlet of the jet system. Gas adjustment is done by Harris Argon 355–2 Flowmeter Regulator. Besides, the Ar gas flow rate is 8 Lit/min (133 cm3.s-1) in all measurements. The length of the plasma jet is recorded by a Casio high-speed Exilim Ex-ZR700 Digital Camera. The voltage and the current are measured by a high voltage probe (TEKTRONIX P6015 1:1000) and a current probe (Rogowski coil)^35^, respectively. The electrical signals are visualized using a TEKTRONIX TDS 2024B oscilloscope (200 MHz). The Optical Emission Spectrometer (OES) with model HR 2000 was used to detect the species in plasma jet. The spectroscopy from the plasma jet was measured at a distance of one centimeter radially and two centimeters longitudinally where the plasma jet hit the surface.

## References

[1] Maho T, Binois R, Brulé-Morabito F, Demasure M, Douat C, Dozias S, Escot Bocanegra P (2021). Anti-bacterial action of plasma multi-jets in the context of chronic wound healing. Appl. Sci..

[2] Bekeschus S, Favia P, Robert E, von Woedtke T (2019). White paper on plasma for medicine and hygiene: Future in plasma health sciences. Plasma Process. Polym..

[3] Busco G, Robert E, Chettouh-Hammas N, Pouvesle J-M, Grillon C (2020). The emerging potential of cold atmospheric plasma in skin biology. Free Radic. Biol. Med..

[4] Vandamme M, Robert E, Lerondel S, Sarron V, Ries D, Dozias S, Sobilo J (2012). ROS implication in a new antitumor strategy based on non-thermal plasma. Int. J. Cancer.

[5] Stancampiano A, Gallingani T, Gherardi M, Machala Z, Maguire P, Colombo V, Pouvesle J-M, Robert E (2019). Plasma and aerosols: Challenges, opportunities and perspectives. Appl. Sci..

[6] Güner MH, Görgülü T, Olgun A, Torun M, Kargi E (2016). Effects of ozone gas on skin flaps viability in rats: an experimental study. J. Plastic Surg. Hand Surg..

[7] Conrads H, Schmidt M (2000). Plasma generation and plasma sources. Plasma Sources Sci. Technol..

[8] Walsh JL, Shi JJ, Kong MG (2006). Contrasting characteristics of pulsed and sinusoidal cold atmospheric plasma jets. Appl. Phys. Lett..

[9] Kim SJ, Chung TH (2016). Cold atmospheric plasma jet-generated RONS and their selective effects on normal and carcinoma cells. Sci. Rep..

[10] Bormashenko E, Grynyov R, Bormashenko Y, Drori E (2012). Cold radiofrequency plasma treatment modifies wettability and germination speed of plant seeds. Sci. Rep..

[11] Khlyustova A, Labay C, Machala Z, Ginebra M-P, Canal C (2019). Important parameters in plasma jets for the production of RONS in liquids for plasma medicine: A brief review. Front. Chem. Sci. Eng..

[12] Laroussi M, Akan T (2007). Arc-free atmospheric pressure cold plasma jets: A review. Plasma Process. Polym..

[13] Li X, Wu J, Jia B, Wu K, Kang P, Zhang F, Zhao N, Jia P, Wang L, Li S (2020). Generation of a large-scale uniform plasma plume through the interactions between a pair of atmospheric pressure argon plasma jets. Appl. Phys. Lett..

[14] Fu W, Zhang C, Nie C, Li X, Yan Y (2019). A high efficiency low-temperature microwave-driven atmospheric pressure plasma jet. Appl. Phys. Lett..

[15] Shashurin A, Keidar M (2015). Experimental approaches for studying non-equilibrium atmospheric plasma jets. Phys. Plasmas.

[16] Robert E, Sarron V, Ries D, Dozias S, Vandamme M, Pouvesle JM (2012). Characterization of pulsed atmospheric-pressure plasma streams (PAPS) generated by a plasma gun. Plasma Sources Sci. Technol..

[17] Breden D, Miki K, Raja LL (2011). Computational study of cold atmospheric nanosecond pulsed helium plasma jet in air. Appl. Phys. Lett..

[18] Seyfi P, Khademi A, Ghasemi S, Farhadizadeh A, Ghomi H (2020). The effect of mixed electric field on characteristic of Ar–N2 plasma jets for TiN surface treatment. J. Phys. D: Appl. Phys..

[19] Guaitella O, Sobota A (2015). The impingement of a kHz helium atmospheric pressure plasma jet on a dielectric surface. J. Phys. D Appl. Phys..

[20] Sands BL, Ganguly BN, Tachibana K (2008). A streamer-like atmospheric pressure plasma jet. Appl. Phys. Lett..

[21] Pinchuk ME, Stepanova OM, Gromov M, Leys C, Nikiforov A (2020). Variation in guided streamer propagation along a DBD plasma jet by tailoring the applied voltage waveform. Appl. Phys. Lett..

[22] Stancampiano A, Chung T-H, Dozias S, Pouvesle J-M, Mir LM, Robert E (2019). Mimicking of human body electrical characteristic for easier translation of plasma biomedical studies to clinical applications. IEEE Trans. Radiat. Plasma Med. Sci..

[23] Akatsuka H (2019). Optical Emission Spectroscopic (OES) analysis for diagnostics of electron density and temperature in non-equilibrium argon plasma based on collisional-radiative model. Adv. Phys. X.

[24] Polo JA, Lakhtakia A (2011). Surface electromagnetic waves: A review. Laser & Photon. Rev..

[25] Kristensson G (1995). Transient electromagnetic wave propagation in waveguides. J. Electromag. Waves Appl..

[26] Robert E, Darny T, Dozias S, Iseni S, Pouvesle J-M (2015). New insights on the propagation of pulsed atmospheric plasma streams: From single jet to multi jet arrays. Phys. Plasmas.

[27] Obradović BM, Ivković SS, Kuraica MM (2008). Spectroscopic measurement of electric field in dielectric barrier discharge in helium. Appl. Phys. Lett..

[28] Bourdon A, Darny T, Pechereau F, Pouvesle J-M, Viegas P, Iséni S, Robert E (2016). Numerical and experimental study of the dynamics of a μs helium plasma gun discharge with various amounts of N2 admixture. Plasma Sources Sci. Technol..

[29] Dozias, S., Pouvesle, J.M., & Robert, E. Comment on ‘Mapping the electric field vector of guided ionization waves at atmospheric pressure’,(2020) Plasma Res. Express 2 025014. *Plasma Res. Express 3*(3), 038001 (2021).

[30] Lietz, A.M., Damany, X., Robert, E., Pouvesle, J. M., & Kushner, M. J. Ionization wave propagation in an atmospheric pressure plasma multi-jet. *Plasma Sources Sci. Technol.* **28**(12), 125009 (2019).

[31] Xiong Z, Kushner MJ (2012). Atmospheric pressure ionization waves propagating through a flexible high aspect ratio capillary channel and impinging upon a target. Plasma Sources Sci. Technol..

[32] Vijayarangan V, Delalande A, Dozias S, Pouvesle J-M, Robert E, Pichon C (2020). New insights on molecular internalization and drug delivery following plasma jet exposures. Int. J. Pharm..

[33] Vijayarangan V, Delalande A, Dozias S, Pouvesle J-M, Pichon C, Robert E (2017). Cold atmospheric plasma parameters investigation for efficient drug delivery in HeLa cells. IEEE Trans. Radiat. Plasma Med. Sci..

[34] Seyfi P, Zahedi S, Shojaei H, Ghomi H (2022). Investigation of the effects of mixed electric field stress on high voltage transformer insulation. Electr. Eng..

[35] Argüeso M, Robles G, Sanz J (2005). Measurement of high frequency currents with a Rogowski coil. Rev. Sci. Instrum.

